# Distinct Neural Modes Carry Information About Attempted Grasp Timing and Force in the Sensorimotor Cortex

**DOI:** 10.1523/JNEUROSCI.0205-26.2026

**Published:** 2026-07-28

**Authors:** G. H. Blumenthal, B. M. Dekleva, C. Gontier, I. C. Gonzalez, J. A. Gonzalez-Martinez, B. M. Yu, A. P. Batista, A. R. Sobinov, L. E. Miller, R. A. Gaunt, M. L. Boninger, S. M. Chase, J. L. Collinger

**Affiliations:** ^1^Rehab Neural Engineering Labs, University of Pittsburgh, Pittsburgh, Pennsylvania 15219; ^2^Department of Physical Medicine and Rehabilitation, University of Pittsburgh, Pittsburgh, Pennsylvania 15213; ^3^Center for Neural Basis of Cognition, Pittsburgh, Pennsylvania 15213; ^4^Department of Neurological Surgery, University of Pittsburgh Medical Center, Pittsburgh, Pennsylvania 15213; ^5^Neuroscience Institute, Carnegie Mellon University, Pittsburgh, Pennsylvania 15213; ^6^Departments of Biomedical Engineering, Carnegie Mellon University, Pittsburgh, Pennsylvania 15213; ^7^Electrical and Computer Engineering, Carnegie Mellon University, Pittsburgh, Pennsylvania 15213; ^8^Department of Bioengineering, University of Pittsburgh, Pittsburgh, Pennsylvania 15213; ^9^Department of Organismal Biology and Anatomy, University of Chicago, Chicago, Illinois 60637; ^10^Department of Neuroscience, Northwestern University, Chicago, Illinois 60208

**Keywords:** force, grasp, human, motor control, motor cortex, somatosensory cortex

## Abstract

Humans perform a variety of complex hand movements to manipulate objects, requiring precise control of changing forces. Understanding the role of sensorimotor cortex and the cortical dynamics underlying these actions is crucial for developing interventions that restore dexterous hand function after injury or disease. In this study, two male individuals with tetraplegia resulting from cervical spinal cord injury attempted a series of isometric grasps. Neural activity was recorded from the motor and somatosensory cortices using intracortical microelectrode arrays while participants attempted to exert a static or ramping force up and down. Despite their inability to execute movement and limited afferent input, the spiking activity in motor and somatosensory cortex was modulated with the task. Within the neural response, we identified independent neural modes—distinct patterns of population-level neural activity that were informative about both the timing and magnitude of the attempted force. Moreover, distinct neural modes were observed during static and dynamic grasping conditions, suggesting independent control schemes for maintaining and changing forces. These modes were related to phases of the task, including the onset, offset, holding periods, as well as increasing and decreasing attempted forces. These results will inform the design of intracortical brain–computer interface (iBCI) systems that can leverage the patterns of grasp and force control evident in sensorimotor cortex during attempted movement to restore dexterous hand function.

## Significance Statement

Restoring dexterous hand function after injury remains a major challenge, partly due to an incomplete understanding of the cortical dynamics underlying grasping and force control. In this study, we investigated neural activity within the motor and somatosensory cortices of individuals with tetraplegia attempting to perform grasps to different target forces with varying temporal profiles. We identified distinct neural modes modulated during specific phases of grasp that encode attempted force information throughout the task. These findings suggest that brain–computer interfaces could leverage these neural modes to restore grasping and force modulation.

## Introduction

Restoration of upper limb motor function is a clinical priority for individuals with cervical spinal cord injury (Anderson, 2004; Simpson et al., 2012; Collinger et al., 2013a; Thorogood et al., 2023). Intracortical brain–computer interfaces (iBCIs) aim to restore arm and hand function by decoding motor intention from the motor cortex, enabling intuitive effector control over multiple degrees of freedom (Hochberg et al., 2012; Collinger et al., 2013b; Wodlinger et al., 2015; Bouton et al., 2016; Ajiboye et al., 2017; Guan et al., 2023). However, achieving dexterous object manipulation, which requires precise force control, remains a major challenge for iBCI systems (Pandarinath and Bensmaia, 2022). This limitation partly arises from an incomplete understanding of the cortical dynamics underlying grasp and force control within the sensorimotor cortex.

Force- and grasp-related activity has been observed in the motor cortex (MC) in both humans (Downey et al., 2018; Rastogi et al., 2020; 2021; Okorokova et al., 2024) and nonhuman primates (Evarts, 1968; Smith et al., 1975; Hepp-Reymond et al., 1978; Cheney and Fetz, 1980; Maier et al., 1993; Oby et al., 2013; Mollazadeh et al., 2014; Menz et al., 2015; Rouse and Schieber, 2016; Okorokova et al., 2024). Early intracortical recordings revealed diverse force-tuning properties at the single-neuron level, where unit activity closely correlates with muscle activity and force output (Evarts, 1968; 1969; Smith et al., 1975; Wannier et al., 1991). At the population level, ensembles of MC neurons are flexibly recruited across diverse motor behaviors, with populations encoding force often overlapping those representing grasp type or other kinematic features. The extent of this overlap remains debated: some studies suggest relative independence of force information (Hendrix et al., 2009; Moreno et al., 2025), while others report interaction between force and kinematic parameters (Degenhart et al., 2011; Intveld et al., 2018; Rastogi et al., 2021). Such overlap may limit the utility of force-related signals during complex behaviors, as evidenced by reduced grasp information during simultaneous movement of the arm during object transport (Downey et al., 2018). Advancing dexterous iBCI control thus requires understanding population-level MC dynamics across varied force conditions, including both static and dynamic force applications.

The MC operates within an interconnected sensorimotor network. The primary somatosensory cortex (SC), for example, is also highly active during grasp (Cavina-Pratesi et al., 2010). While much of this activity reflects peripheral sensory input, corticocortical communication from MC to SC also contributes, consistent with the dense bidirectional connectivity between these brain areas (Huber et al., 2017; Umeda et al., 2019; Rolls et al., 2023). Under the “forward model” framework (Wolpert et al., 1995), MC sends an efference copy of motor commands to SC, which modulates sensory input to distinguish self-generated from external stimuli (Straka et al., 2018). A landmark study showed that subpopulations of SC neurons in monkeys could differentiate between active and passive reaching with some firing prior to movement onset, including both excitatory and suppressive efference copy signals from MC (London and Miller, 2012). Furthermore, kinematic information can be decoded from SC during imagined movements, even without peripheral input (Jafari et al., 2020). Yet, efference copy signaling related to force application remains poorly understood, complicated by concurrent proprioceptive and tactile feedback during natural motor actions.

Given this complexity, the present study aimed to characterize neural population activity in MC and SC during attempted grasping and to identify the types of information encoded. We employed a population analysis approach that lends explanation to the diversity of results previously reported in single-neuron studies. Two individuals with tetraplegia resulting from cervical spinal cord injury attempted isometric grasps at varying force targets and rates of force application while neural activity was recorded from MC and SC using intracortical microelectrode arrays. We identified distinct neural modes in both regions that encoded specific phase- and attempted force-related information. These findings advance understanding of sensorimotor population dynamics and have implications for developing iBCIs capable of restoring dexterous functional grasp in individuals with paralysis.

## Materials and Methods

### Experimental design

**Figure 1.**
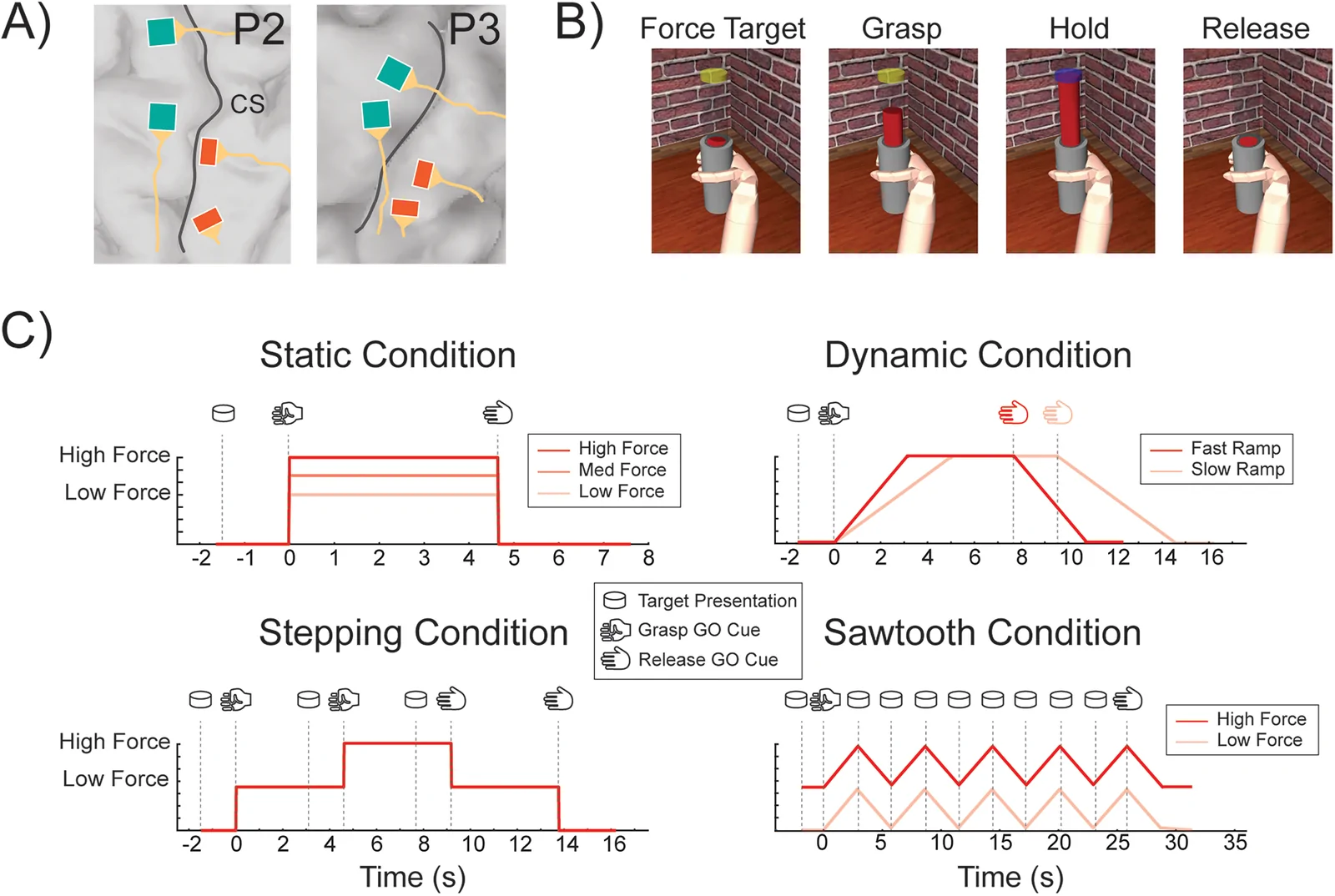
Experimental Design: ***A***, Location and orientation of the MC (green) and SC (red) arrays in the left motor and somatosensory cortex. CS is the location of the central sulcus. ***B***, Depiction of a virtual grasping task at each phase, including a force target presentation phase, a grasp phase, force holding phase, and a release phase. ***C***, Examples of force profiles and task timing for four different grasping conditions. Static conditions consisted of an instantaneous grasp for a target force, a holding period, and an instantaneous release. Dynamic conditions included a ramping phase over a variable time period to a target force, a holding period, and a corresponding ramping down phase to release. Ramp durations included 1.5, 3.0, 4.5, and 6.0 s (two shown) and varied across trials to produce different rates of attempted force change. More complex conditions included a static force stepping condition and a dynamic sawtooth condition across two different force ranges.

Data were collected as part of an ongoing clinical trial (NCT01894802) of an intracortical sensorimotor brain–computer interface (iBCI) conducted under an Investigational Device Exemption (IDE) granted by the U.S. Food and Drug Administration and approved by the University of Pittsburgh Institutional Review Board. Two individuals (P2 and P3) with cervical spinal cord injury (SCI) participated in the study and provided informed consent prior to any experimental procedures. For each participant, two intracortical microelectrode arrays (Blackrock Neurotech, Salt Lake City, UT) were implanted in the hand and arm areas of motor cortex on the surface of the precentral gyrus, which we refer to here as MC (Glasser et al., 2016; Willett et al., 2020; P2: two 88-channel arrays; P3: two 96-channel arrays). Two additional 32-channel microelectrode arrays were implanted in the hand area of somatosensory cortex (SC; Fig. 1*A*). P2 was a 28-year-old male (at the time of implant) with tetraplegia caused by a C5 motor/C6 sensory incomplete ASIA B spinal cord injury that was sustained ∼10 years prior to microelectrode implantation surgery. Data were collected 8–9 years after implant. P3 was a 28-year-old male (at the time of implant) with tetraplegia caused by a C6 ASIA B spinal cord injury that was sustained 12 years before implantation surgery. Data were collected ∼4 years after implant. Both participants have some residual upper arm and wrist motor function and some residual sensation in their hands. They do not have any hand-related motor function and therefore could not execute grasps. Instead, all grasps were attempted by participants.

During an experimental session, participants remained in a position such that the weight of their right arm was supported against gravity. A virtual environment built within the MuJoCo physics simulator (https://mujoco.org/) was displayed on a screen in front of them and, along with audio cues, used to prompt participants to the timing of the task (Fig. 1*B*). All kinematic and force applications in the simulator were predefined and controlled automatically without participant input or control. In other words, participants were instructed to “follow along” with the actions depicted in the simulation.

Whenever force was applied to the gray cylinder, a smaller red cylinder would rise out of it to a height that represented the currently attempted force. All tasks consisted of (1) a force target presentation phase: The force target was represented by a yellow disk placed above the gray cylinder at a specific height for 1.5 s. (2) A grasp phase initiated by an audio GO cue: Virtual force was applied to the gray cylinder and the red cylinder would rise to a height representing the attempted force. (3) A hold phase: When the target force was reached, the force target disk would turn blue. After the specified length of time, the target-force disk would either move to a new height (indicating a new target force) or disappear (indicating that the object should be released) 1.5 s before another audio cue, which cued the (4) release phase, when the participants would release their grasp.

Four different grasping conditions were sampled—static, dynamic, stepping, and sawtooth (Fig. 1*C*). Static grasping conditions consisted of an instantaneous grasp to a specific force level (high, medium, or low), a period of maintaining (holding) the target force, and an instantaneous release. To help participants conceptualize force levels, we verbally described the low-, medium-, and high-force targets as equivalent to using the attempted force required to grasp and hold a tomato, orange, and a can of soup, respectively, without the object slipping (Downey et al., 2018). Dynamic grasping conditions consisted of attempted force ramping over a set period (1.5–6 s) until a high force target was reached, a holding period, and a corresponding ramp-down until full release. Constraining the ramp to variable time windows effectively produced different conditions where the rate of ramp was varied. The stepping condition combined sequential static attempted force grasps within the same trial. Specifically, a low-force static grasp, followed by a high-force static grasp, a final low-force static grasp, and release. Finally, the sawtooth condition combined several sequential ramping periods without holding periods. During each session, at least 20 trials of each condition were collected, and at least six sessions were carried out per participant.

Both participants had prior experience with grasp and force-modulation tasks at the time of data collection. P2 previously performed attempted grasping of four objects with different force requirements, with offline classification of MC activity reaching 69% accuracy (chance, 25%) and dropping to near chance (29%) during passive observation of the same objects (Downey et al., 2018). Both P2 and P3 also performed overt (using residual wrist function) and imagined isometric wrist extensions to graded force targets, each reliably modulating motor cortex population activity in a force-appropriate manner (Dekleva et al., 2024). The majority of prior closed-loop iBCI work in both participants involved decoding of grasp kinematics (e.g., hand open/close) rather than force. Continuous online decoding of grasp force has historically been challenging (Dekleva and Collinger, 2025), and prior online force-decoding experience in these participants has been limited to simple two- and four-level static tasks. The dynamic ramp-and-hold paradigm used in the present study was novel for both participants.

### Electrophysiology and data recording

Neural data were collected from MC and SC via two percutaneous pedestal connectors using NeuroPlex-E headstages connected to two synced Neural Signal Processors (Blackrock Neurotech). Signals were hardware filtered between 0.3 and 7,500 Hz, sampled at 30,000 Hz, and digitally filtered with a 1st order 750 Hz Butterworth high-pass filter. Spikes exceeding a threshold of −4.5 root mean square (RMS) of the signal on each channel were counted and binned into 20 ms bins.

### Analyses and statistics

Neural data analysis was performed separately for recordings from MC and SC. Spike counts were square root transformed and convolved with a noncausal Gaussian kernel (*σ* = 200 ms) to obtain a smoothed estimate of firing rates for each channel. First, we examined responses on individual channels, and then we characterized the neural dynamics observed at a population level.

**Figure 2.**
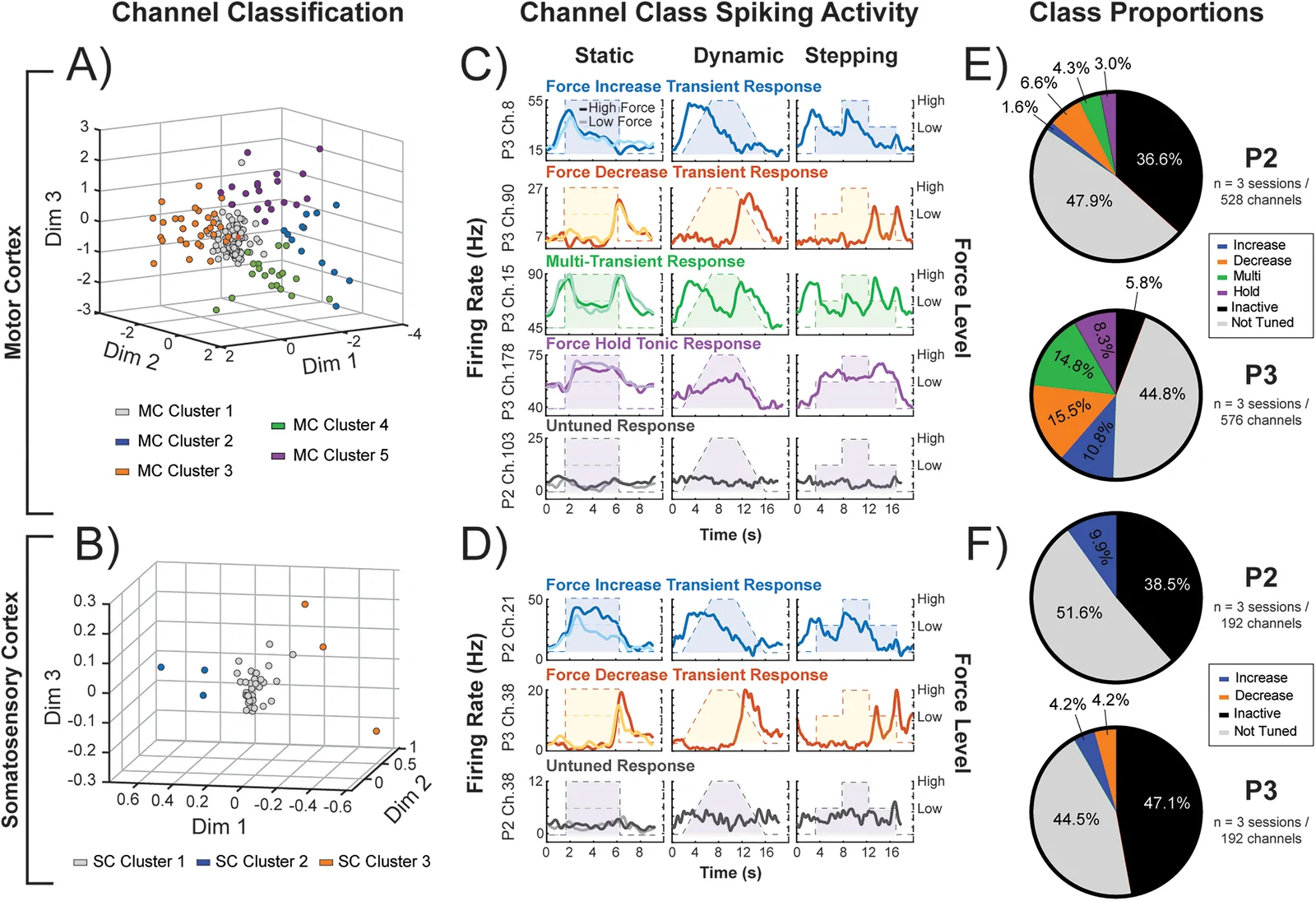
Spiking activity patterns on single channels: ***A***, ***B***, *K*-means clustering of channel spiking activity during all grasp conditions in (***A***) motor cortex (MC) and (***B***) somatosensory cortex (SC). Each dot represents a channel. An example session from P3 is shown here. *K*-means clustering was performed on the first three factors of each channel's binned firing-rate feature vector (*k* = 5 for MC in both participants; *k* = 2 for P2 SC and *k* = 3 for P3 SC; see Materials and Methods). ***C***, ***D***, Example channels of trial-averaged responses from each cluster across each grasping condition in (***C***) motor cortex and (***D***) somatosensory cortex. Dotted lines represent the force profile in each condition. Classes were defined for each cluster describing their response profile. Example classes from both participants (P2 and P3) are shown here. ***E***, ***F***, Proportion of channels in each class for (***E***) motor cortex and (***F***) somatosensory cortex. Proportions are expressed relative to all recorded channels in each region (including untuned channels).

To analyze responses on single channels, all data were grouped by attempted force and rate conditions. Every trial was time-aligned within condition to the onset of grasp. For each channel, spike rates were then averaged across trials within condition. Low-firing channels (<1 Hz) were labeled “Inactive” and removed from any further single-channel analyses. Upon visual inspection, we observed different channel-specific temporal profiles in the trial-averaged firing rates, including transient activations during onset and offset of grasp as well as tonic activity during static attempted force periods. To classify channels into different response types, we first performed factor analysis (FA) on the averaged spike rates, with time as features and channels as observations. To reduce the number of time bins (∼500–1,750 per trial) while minimizing temporal dependence, we divided the trial into nonoverlapping 1 s windows and averaged the spike rates within each window. This approach effectively downsampled the data to ∼10–20 features per trial, depending on trial duration. Note that this 1 s binning defines the feature space used to cluster channels by their response profiles and does not affect the temporal resolution of the underlying firing-rate estimates, which is set by the 200 ms Gaussian smoothing described above (see example single-channel responses in Fig. 2*C*,*D*).

*K*-means clustering was then performed on the three factors which explained the most variance of the data. A reasonable number of clusters to use were determined by finding the inflection point of the curve generated when calculating the combined sum of the within-cluster sum of point-to-centroid distances across 1–10 clusters. We used five clusters for both P2 and P3 for MC data, two for P2 SC data, and three for P3 SC data. The channels associated with each cluster were labeled as a “class” according to their shared qualitative firing patterns. Additionally, all channels were visually examined. Some channels that were on the border of two or more clusters in factor space were manually assigned to a different class if it was determined that a channel was more representative of that class across all conditions. This process was performed using data from three different sessions per participant, treating each channel within a session as an independent observation. Thus, our analysis captures the distribution of observed channel response types across sessions, rather than the activity of longitudinally tracked channels. Finally, the proportion of channels within each class was calculated and compared between participants using a chi-square contingency table.

For each session, we first estimated the dimensionality of the neural population activity using fourfold cross-validated FA (Sadtler et al., 2014). For each tested dimensionality (1–30), an FA model was trained on 75% of the data and its log-likelihood evaluated on the remaining 25%. This process was repeated across folds, and the dimensionality that maximized cross-validated log-likelihood was taken as the session's estimated dimensionality. These results informed our choice to set the latent dimensionality at 20 for subsequent analyses, which captured the majority of explainable variance across sessions while allowing consistency across participants. We then applied FA with 20 factors to the trial-averaged data from each session, followed by a varimax rotation to improve interpretability of temporal features. The rotation was performed on the baseline-centered factors, where the baseline was defined as the averaged response in a 200 ms window beginning 1.5 s before the onset of grasp, and the resulting components were then ranked by decreasing explained variance (Dekleva et al., 2024). Finally, to align latent spaces across multiple sessions for each participant, we applied generalized Procrustes analysis (GPA), which finds a linear transformation that minimizes differences between session-specific latent trajectories and a consensus configuration (Gower, 1975). GPA included an isotropic scaling factor for each session, estimated as part of the Procrustes transformation, and the consensus shape was rescaled to the magnitude of the initial reference dataset after convergence. Across sessions and arrays in both participants (*n* = 24), per-session scaling factors had a mean of 0.92 and ranged from 0.71 to 1.29. Because this scaling is isotropic, it is applied uniformly across all factors within a session and therefore preserves within-session relative variance across modes. The resulting aligned factors (neural modes) represent dominant, interpretable patterns of population covariation (Gallego et al., 2017 ). Modes were then labeled by identifying which ones were most strongly modulated during specific phases of the grasp. For single-trial analyses, we projected each trial's factor scores into the same rotated latent space used for condition averages. Specifically, the orthogonal varimax rotation matrix *Q*, estimated from the condition-averaged factor trajectories, was applied to the factor time series of each individual trial, resulting in single-trial trajectories in a common factor basis. For each trial and mode, we computed the moving sum of squared factor scores within a fixed 200 ms window and divided by that mode's total per-trial variance. We then averaged these time courses across trials (by condition) and across sessions after GPA alignment. A mode's label (onset, hold, offset, increase, decrease) was assigned by the phase window in which its condition-averaged variance fraction attained its maximum. Because windows overlap, values are not additive across time—we report peaks and phase-integrated fractions for interpretation.

To understand timing relationships between modes, we quantified the latencies between transient peaks and the time points of when relevant modes reached 25, 50, 75, and 90% of their peak modulation during each trial. Mode activations were normalized per trial to their own maxima so that percent thresholds were comparable across trials. Mode-specific search windows were centered around onset and release GO cues ±1.5 s to identify transient peaks, force holding, and force ramping windows. All landmarks were restricted to these windows. We calculated the timing between transient and holding- and ramping-related mode landmarks such that the sign convention yields positive values when the mode modulation landmark occurs after the transient. To analyze differences in the timing of neural mode modulation across force conditions (i.e., low vs high) and modulation levels (i.e., 25, 50, 75, 90%), we performed a two-way ANOVA on the data to look for main effects, and a post hoc Šidák correction to control for multiple-comparisons familywise error.

To gain an understanding of force representation in MC and SC, we fit a naive Bayes classifier (Downey et al., 2018) to each time point across static force conditions using two and three force levels and dynamic conditions (without a static phase) using two force levels. A new model was fit to every classification window, defined using a sliding window of 200 ms centered on each time point. GPA-aligned data were used as input, averaged across time within each window. Each classification window was leave-one-out cross-validated, and the overall percent correct prediction was calculated. This resulted in a condition-classification curve across the trials. Classification ability is a metric that we defined as the area under this curve bounded by chance levels and relevant phase time points.

Encoding of force rate in the cortex was investigated by performing classification of four ramping conditions (1.5, 3.0, 4.5, 6.0 s ramps to a high-force target) using linear discriminant analysis (LDA). We slid a fixed-length window (250 ms, step 50 ms) over each trial. For every window we computed: the mean latent factor vector (across time within the window) and the mean force within the window. The mean force was quantized into bins of width 0.1. To ensure strict force matching and to avoid multiple correlated samples per trial, we enforced one window per trial per force bin. Each retained window was labeled by the ramp duration (four-way classification; one class per instructed ramp duration). LDA was performed with uniform priors, separately for ramp-up and ramp-down windows. Fivefold cross-validation (CV) was performed and was group-aware: folds partitioned trials, and all windows from a trial/group appeared in exactly one-fold. Within each fold, features were *z*-scored using training statistics and any zero-variance columns were dropped. For each force bin, we pooled predictions from all CV test folds to obtain a single proportion correct (chance = 0.25). A bin-wise accuracy curve was calculated with a Wilson 95% binomial confidence interval. Bin-wise permutation *p* values were computed by repeating the grouped CV after shuffling duration labels within CV groups (thus preserving force matching, window counts, and train/test splits). The *p* value is the exceedance probability of the observed accuracy under the null (5,000 permutations). Because multiple bins are tested, we applied Benjamini–Hochberg FDR correction (*q* = 0.05) separately for ramp-up and ramp-down panels. To probe shared dynamics between MC and SC, we performed canonical correlation analysis (CCA) on the per-trial latent factor projections described above. For each participant, single-trial MC and SC factor trajectories (20 modes per area) were pooled across sessions over the temporally aligned trial epochs and concatenated in time, yielding paired matrices of dimension T × 20 for MC and SC. CCA (MATLAB canoncorr) returned canonical weights A (MC) and B (SC), the canonical variates U = MC·A and V = SC·B, and per-dimension paired correlations *r*. Canonical dimensions with *r* > 0.3 (a moderate effect-size threshold) were retained as high-effect dimensions for the analyses below.

To characterize the shared subspace, we computed Spearman correlations between each high-effect canonical variate and three trial-aligned regressors: (1) a sustained force-command regressor (per-bin attempted force), (2) a grasp-transient regressor (boxcar of width ±500 ms centered on both the grasp-onset and release cues), and (3) a force-transition regressor (smoothed |dF / dt|, ∼100 ms FWHM). For each high-effect dimension, we averaged the absolute Spearman correlation across the MC variate and the SC variate to produce a symmetric per-pair tuning summary. To calibrate against chance, we computed a per-participant, per-regressor permutation null by within-trial circular shift of the regressor (300 permutations, pooled across high-effect dimensions); the 95th percentile of the resulting distribution defined the participant-specific noise floor. Channel-loading composition for each high-effect dimension was summarized by aggregating the absolute weights according to each channel's single-channel response class (onset, offset, increase, decrease, hold-high, hold-low, other).

To test for directional asymmetry in MC–SC coupling, we asked whether trial-by-trial fluctuations of the shared dynamics appear earlier in one area than the other. Trial-averaged structure shared between MC and SC can produce a spurious near-zero-lag correlation that masks any true directional lag, so we first subtracted the per-condition trial-averaged time course from each factor (analogous to noise correlation analysis) and then applied the canonical weights to these residuals. For every MC × SC mode pair, we computed the cross-covariance of the residual variates over a ±500 ms search window and recorded the peak lag and the signed cross-covariance at that lag (peak *r*). By convention, negative peak lag indicates MC leads SC. The full mode-pair matrix was also generated. We restricted the directional analysis to pairs with nontrivial residual coupling (|peak *r*| ≥ 0.10) to avoid interpreting noise-driven lags and tested the lag-sign distribution against chance using a two-sided binomial sign test, treating each mode pair as one observation.

## Results

### Single channels are tuned to specific phases of the grasp

In studying single channel responses during grasping behavior, we observed channel tuning to specific phases of the grasp in both MC and SC. Across three sessions from each participant (total channels: P2: MC = 528, SC = 192; P3: MC = 576, SC = 192), spike rates for a given channel were averaged across all trials within each condition, and *K*-means clustering was used to group channels with similar firing patterns (Fig. 2*A*,*B*). Per-channel firing-rate heatmaps for both regions, grouped by response class and condition, are provided in Figure S1. As channel response patterns cannot be stably tracked across sessions, we analyzed each channel/session independently. Reported proportions therefore reflect the distribution of observed response classes across three sessions per participant, not unique neurons. In MC, upon visual inspection, these distinct firing patterns represented transient responses during increases in force, transient responses during decreases in force, transient responses during any change in force (“multi-transient”), or tonic responses during holding periods of constant force (Fig. 2*C*). Similar but fewer classes were identified in SC, including transient responses during increases in force and transient responses during decreases in force (Fig. 2*D*). We did not identify any SC channels with the multitransient or tonic hold responses that were identified in MC, however, the channels with transient responses during increases in force tended to have a longer tail that extended into the holding periods (Fig. 2*D*).

In P2, the total proportion of MC channels with modulated firing patterns was 15.5%, with each class accounting for between 1.6 and 6.6% of total channels. In P3, this proportion was 49.4%, with each class accounting for between 8.3 and 15.5% of total channels (Fig. 2*E*). This difference is likely due to the durations of the implants with fewer channels in P2 recording neural signals at >8 years postimplant. Excluding channels considered inactive and not tuned, we found no significant difference in proportions of classes between participants [χ^2^ (3, *n* = 361) = 7.43, *p* = 0.059], suggesting a similar distribution of tuned channel classes across participants. Overall, fewer SC channels were modulated to the task (Fig. 2*F*). Because we did not identify any decrease-specific channels in SC for P2, no additional statistics were performed on SC class proportions. In summary, we observed that both MC and SC contained channels with transient responses tuned to increases and decreases in force. However, only MC contained multitransient responses modulated by both increases and decreases in force, as well as tonic responses during the hold phase. These results indicate that, while both MC and SC demonstrate multiunit task-related tuning, MC exhibits a broader repertoire of force-related response patterns than SC, consistent with its primary role in initiating and sustaining grasp forces.

### Distinct MC neural modes capture phase-specific activity during grasping

Having demonstrated the variety of individual channel responses, we went on to analyze the population response of the sensorimotor cortex during grasping. We did this by examining the dominant, interpretable patterns of population covariance, or neural modes, from both the MC and SC during the attempted behavior (see Materials and Methods). Unlike per-channel labeling, population activity can capture coherent modes from groups of weakly modulated neurons and yields orthogonal dimensions that partition shared variance into distinct components (Cunningham and Yu, 2014). In both participants, individual neural modes were consistently modulated during specific phases of the grasp (e.g., onset, hold, release), and this phase-locked modulation was preserved across all grasping conditions.

**Figure 3.**
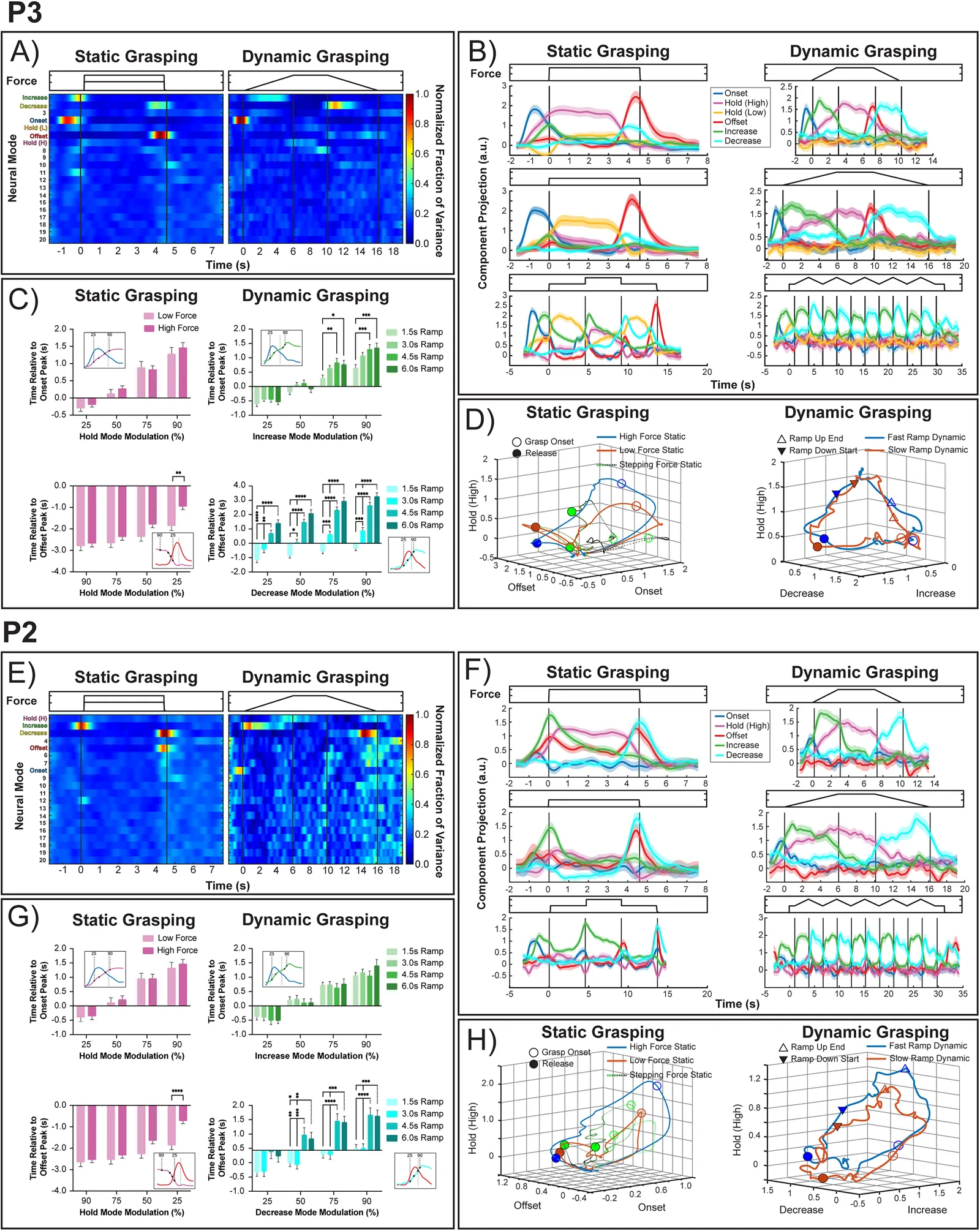
MC neural modes for participant P3 (***A–D***) and P2 (***E–H***): ***A***, ***E***, Heatmaps show temporal concentration of variance for each mode. Color values are normalized to the global maximum across all modes, such that 1 represents the strongest hotspot observed. Because windows overlap, values across time are not additive. ***B***, ***F***, Trial-averaged neural modes during static and dynamic grasping conditions. Modes represent GPA-aligned data from three sessions per participant. Shaded regions represent the 95% CI across trials (*N* = 60), pooled across three sessions after GPA alignment. ***C***, ***G***, Mean latency (±SEM) of when the holding-related mode (left) and the increase/decrease modes (right) reached 25–90% of their total modulation relative to the peak of the onset transient (top) and the offset transient (bottom; ****p < 0.0001, ***p < 0.001, **p < 0.01, *p < 0.05). The *x*-axis indicates the threshold (percentage of total mode modulation) used to define each latency landmark, and the *y*-axis indicates the time at which that threshold is reached relative to the corresponding transient peak. The inset in each subpanel schematically illustrates the latency landmark being measured: the trace with the dots shows the mode whose modulation defines the threshold, and the second trace shows the reference transient against which the latency is measured. The dots indicate the timepoints of the 25, 50, 75, and 90% modulation thresholds. Dashed significance bars indicate the group that all other comparisons are made against during post hoc analysis. ***D***, ***H***, Neural data projected along the onset, offset, and hold (high) dimensions during static grasping (left) and along the increase, decrease, and hold (high) dimensions during dynamic grasping (right).

In MC, the top 10 neural modes captured ∼90.4% of the variance explained in P3, and ∼89.7% of the variance explained in P2, whereas the top 3 neural modes captured ∼43.4 and 61.3% of the variance in P3 (Fig. S2*A*) and P2 (Fig. S2*C*), respectively. Labels were assigned to modes based on where each mode's fractional variance peaked relative to task phases (Fig. 3*A*,*E*). In MC for P3, we identified six task-relevant neural modes, which captured the following responses: a grasp onset-related transient component, a grasp offset-related transient component, a grasp hold-related static component modulated during high forces, a grasp hold-related static component modulated during low forces, a component modulated during increasing forces, and a component modulated during decreasing forces (Fig. 3*B*).

Largely similar task-relevant modes were observed in P2 (Fig. 3*F*), but their relative engagement differed from P3. The hold-related static component that showed clear modulation at low forces in P3 was not evident in P2. In P2 during static grasping, the force increase- and decrease-related modes were more prominent than the onset-related mode, whereas in P3 the onset-related mode was the dominant component during this phase. During dynamic grasping, the offset-related mode in P2 showed only weak deviations from baseline, in contrast to the pronounced offset-related modulation observed in P3. This difference may reflect differences in ramp-down strategy between participants. The offset-related mode captures rapid, release-like transitions from held force toward zero—an event that is temporally discrete in static grasping but gradual in dynamic grasping. P3 may have initiated dynamic ramp-downs with a sharper decrease than P2, engaging the offset-related mode, whereas P2's more gradual ramp-down may have left it unmodulated.

By comparing the time when the hold and ramp-related modes reached specific modulation levels (25, 50, 75, or 90% of its total modulation) relative to the peaks of the onset and offset transients, we found distinct timing relationships for static versus dynamic grasping (Fig. 3*C*,*G*). In both participants, the hold mode reliably followed onset transients (top left panel) and preceded offset transients (bottom left panel) during static grasping. The onset → hold latencies were not different across force levels for either P3 (Force Cond *F*_(1,472)_ = 0.9920, *p* = 0.3198; Mod Level *F*_(3,472)_ = 58.88, *p* < 0.0001; Interaction *F*_(3,472)_ = 0.3306, *p* = 0.8033) or P2 (Force Cond *F*_(1,672)_ = 0.4457, *p* = 0.5046; Mod Level *F*_(3,672)_ = 48.41, *p* < 0.0001; Interaction *F*_(3,672)_ = 0.07569, *p* = 0.9731), indicating that the timing of grasp onset into sustained holding activity in MC is force invariant. We also looked at the end of the holding period when the grasp transitions from hold to release. An overall timing difference of the hold → offset latencies across force conditions was seen in both P3 (Force Cond *F*_(1,472)_ = 12.72, *p* = 0.0004; Mod Level *F*_(3,472)_ = 19.51, *p* < 0.0001; Interaction *F*_(3,472)_ = 1.644, *p* = 0.1783) and P2 (Force Cond *F*_(1,664)_ = 17.42, *p* < 0.0001; Mod Level *F*_(3,664)_ = 21.96, *p* < 0.0001; Interaction *F*_(3,664)_ = 3.510, *p* = 0.0151) with post hoc analyses revealing a difference only at the 25% level for both participants (bottom left panel). In both cases, the hold component decreased to 25% of its peak value earlier for the low force condition. However, this difference at lower modulation levels may just be due to the low overall modulation of the holding-related mode at low forces.

During dynamic trials, the ramping window, or rate of ramp, was varied across conditions but maintained within each trial until the same force target was achieved across conditions. The timing between transients and ramp-related modes was dependent on ramp duration but showed evidence of a plateau for longer ramps. For P3, both the onset → increase (Ramp Cond *F*_(3,1088)_ = 9.45, *p* < 0.0001; Mod Level *F*_(3,1088)_ = 144.2, *p* < 0.0001; Interaction *F*_(9,1088)_ = 1.319, *p* = 0.2224; Fig. 3*C*, top right panel) and offset → decrease (Ramp Cond *F*_(3,1029)_ = 162.9, *p* < 0.0001; Mod Level *F*_(3,1029)_ = 34.16, *p* < 0.0001; Interaction *F*_(9,1029)_ = 1.165, *p* = 0.3142; Fig. 3*C*, bottom right panel) comparisons showed an overall effect of ramp duration, where in general, the increase and decrease modes peak later in time relative to their respective transients as ramp durations increase. For P2 the effect of ramp duration was not significant for onset → increase (Ramp Cond *F*_(3,944)_ = 0.6585, *p* = 0.5777; Mod Level *F*_(3,944)_ = 103.4, *p* < 0.0001; Interaction *F*_(9,944)_ = 0.4634, *p* = 0.8994; Fig. 3*G*, top right panel), but it did reach significance for offset → decrease (Ramp Cond *F*_(3,869)_ = 32.79, *p* < 0.0001; Mod Level *F*_(3,869)_ = 23.33, *p* < 0.0001; Interaction *F*_(9,869)_ = 0.5254, *p* = 0.8568; Fig. 3*G*, bottom right panel), where shorter decrease ramps reached modulation thresholds earlier than longer ramps, similar to P3. Post hoc comparisons revealed that these differences were driven primarily by the shorter ramps (1.5 and 3.0 s), whereas the relative latencies for the longer ramp conditions (4.5 and 6.0 s) were statistically similar. Thus, the increase and decrease modes appear to be rate-dependent during shorter ramps but saturate during longer ramp durations, consistent with a bounded sensitivity of the observed MC response to ramp speed. Together, these analyses demonstrate that MC transient and sustained/ramp modes are coordinated in a task-specific manner: the timing of the “handoff” between transient (onset and offset) and holding-related modes are independent of force level, while the timing of the “handoff” between transient and ramping-related modes scale with rate of attempted force, especially with shorter ramps. However, with longer ramps, ramping-related modes reach maximal modulation at similar time points relative to transient modes, independent of rate.

Projections of the high- and low-force static grasping conditions onto the most prominent neural modes revealed a visible separation along the high force-specific hold-related dimension (Fig. 3*D*,*H*). This separation was consistent across both participants, supporting the idea that attempted absolute force level is robustly encoded in dedicated hold-related modes. Quantitative support for the separability of force conditions is provided by the force classification analysis described in Figure 5*A*,*B*. Similar force condition separation was observed when projecting the same data along the low force-specific hold-related dimension. In contrast, fast and slow (3 and 6 s ramp duration) dynamic grasping conditions traced largely overlapping trajectories along the force-increase and force-decrease modes. This overlap suggests that the rate of force change was not strongly reflected in the low-dimensional MC trajectories we observed. Taken together, the latency analysis (Fig. 3*C*,*G*) indicates that the temporal coordination between onset/offset transients and the increase/decrease modes varies with ramp kinematics, whereas the trajectory plots (Fig. 3*D*,*H*)—restricted to the increase–decrease–hold subspace—show broadly overlapping ramp trajectories during the ramp itself, suggesting limited separability of ramp conditions in these dimensions once the ramp is underway.

### SC neural modes are modulated during attempted grasp without afferent input

**Figure 4.**
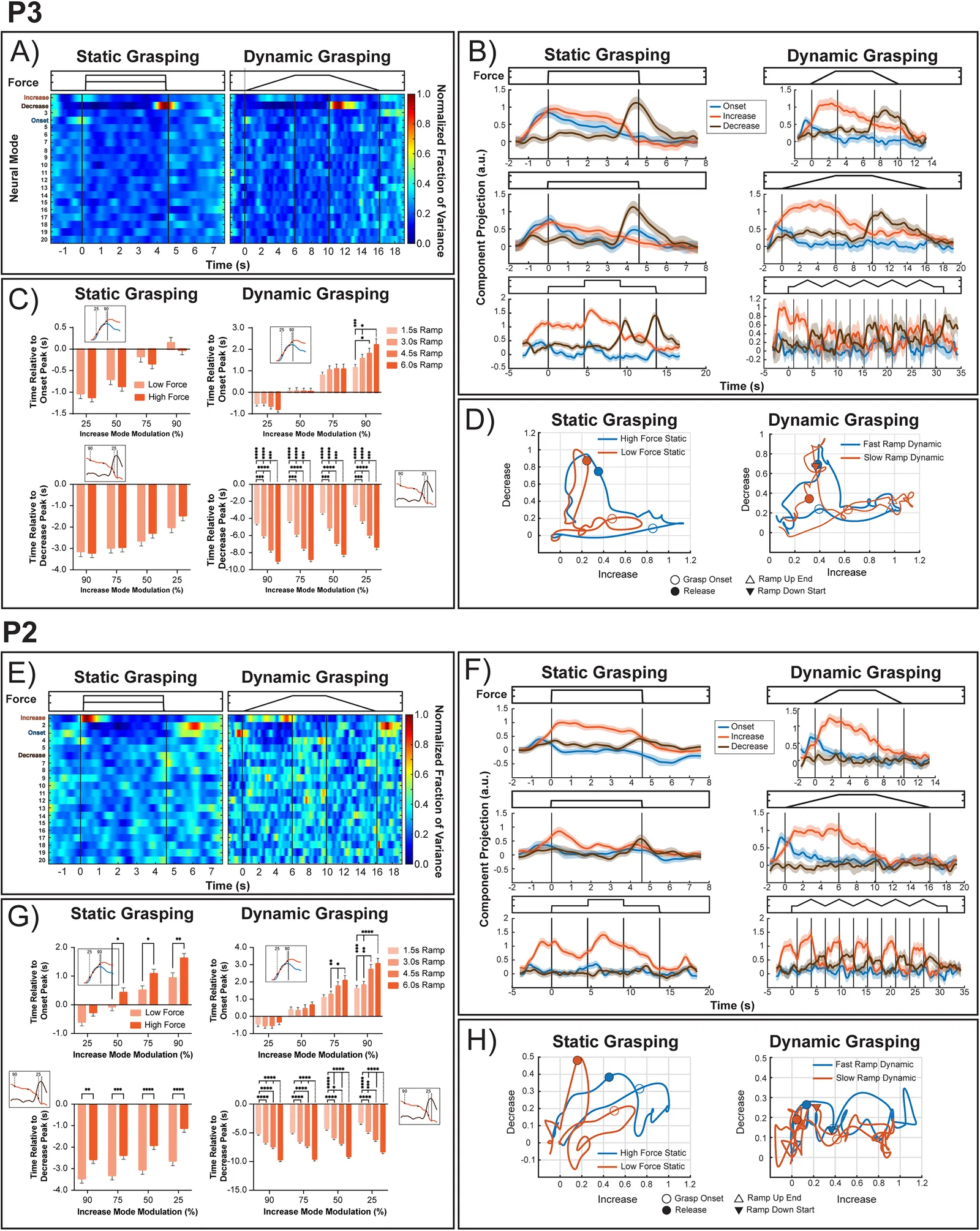
SC neural modes: ***A***, ***E***, Heatmaps show temporal concentration of variance for each mode. Color values are normalized to the global maximum across all modes, such that 1 represents the strongest hotspot observed. Because windows overlap, values across time are not additive. ***B***, ***F***, Trial-averaged neural modes during static and dynamic grasping conditions. Modes represent GPA-aligned data from three sessions per participant. Shaded regions represent the 95% CI across trials (*N* = 60), pooled across three sessions after GPA alignment. ***C***, ***G***, Mean latency (±SEM) of when the increase mode reached 25–90% of its total modulation relative to the peak of the onset transient (top) and the decrease mode (bottom; ****p < 0.0001, ***p < 0.001, **p < 0.01, *p < 0.05). Dashed significance bars indicate the group that all other comparisons are made against during post hoc analysis. ***D***, ***H***, Factor projections along the increase and decrease dimensions during static (left) and dynamic (right) grasping. As in Figure 3*C*,*G*, the *x*-axis in ***C*** and ***G*** indicates the threshold (percentage of total mode modulation) used to define each latency landmark, and the *y*-axis indicates the time at which that threshold is reached relative to the corresponding transient peak; colored bars represent ramp-duration conditions (1.5, 3.0, 4.5, and 6.0 s). The inset in each subpanel schematically illustrates the latency landmark being measured: the trace with the dots shows the mode whose modulation defines the threshold, and the second trace shows the reference transient against which the latency is measured. The dots indicate the timepoints of the 25, 50, 75, and 90% modulation thresholds. ***D***, ***H***, Factor projections in the two-dimensional subspace spanned by the increase and decrease SC modes; trial counts match Figure 3 (*N* = 60 per condition after GPA alignment).

Despite a lack of somatosensory input during attempted motor behavior, we observed task-related modulation of neural modes during grasping in SC, similar to what we observed in MC. Overall, dimensionality was lower than in MC: the top 3 modes explained ∼66.3% of the variance in P3 and ∼62.0% of the variance in P2 (Fig. S3*A,C*), whereas the top 10 neural modes explained ∼88.0 and ∼86.4%, respectively (however, this may be due to fewer recording electrodes in SC—see Materials and Methods). Labels were assigned based on fractional variance peaks relative to task phases (Fig. 4*A*,*E*). In both participants, three task-relevant neural modes were identified: a grasp onset-related mode, a force-increase mode, and a force-decrease mode (Fig. 4*B*,*F*). Compared with MC, the repertoire of SC modes was narrower. There was no clear hold-related mode or offset-related transient. Instead, the increase mode displayed a slow decay that extended into the hold period, suggesting that SC may indirectly represent sustained force levels through prolonged increase-related activity rather than via a dedicated tonic “hold” mode. P3 showed robust modulation of both increase and decrease-related modes, while P2 showed much weaker modulation of the decrease mode during dynamic grasping.

Latency analyses during static grasping compared the onset transient peak with the percent modulation of the increase mode (Fig. 4*C*,*G*, top left). In P3, the onset peak and the 90% level of the increase mode occurred close in time, indicating that the increase mode was recruited almost immediately following grasp onset during static grasping. While an overall difference in force condition was observed (Force Cond *F*_(1,472)_ = 3.872, *p* = 0.0497; Mod Level *F*_(3,472)_ = 46.12, *p* < 0.0001; Interaction *F*_(3,472)_ = 0.07703, *p* = 0.9724), post hoc analysis revealed no significant differences between force levels at any modulation level. In P2, in contrast, the increase mode rose more slowly, with the 90% level reached noticeably later (up to 1.5 s) than the onset peak. Moreover, onset → increase latencies were significantly later for the high force condition compared with low force (Force Cond *F*_(1,672)_ = 31.30, *p* < 0.0001; Mod Level *F*_(3,672)_ = 64.32, *p* < 0.0001; Interaction *F*_(3,672)_ = 0.6185, *p* = 0.6032), suggesting that in P2, phase transitions during static force conditions in SC were partially modulated by force.

We next measured when the increase mode reached 25–90% modulation relative to the peak of the decrease mode (Fig. 4*C*,*G*, bottom left). Across participants, the increase mode's 25–90% landmarks occurred before the decrease peak (i.e., negative latencies), consistent with a slow decay of force increase-related activity that extends into the hold period but subsides ahead of release. In P3, no effect of force was found (Force Cond *F*_(1,466)_ = 2.199, *p* = 0.1388; Mod Level *F*_(3,466)_ = 18.18, *p* < 0.0001; Interaction *F*_(3,466)_ = 0.9091, *p* = 0.4365) suggesting that the relative timing of the increase and decrease components was consistent in both force conditions. In P2, however, these relative latencies differed significantly across force levels (Force Cond *F*_(1,659)_ = 78.54, *p* < 0.0001; Mod Level *F*_(3,659)_ = 15.61, *p* < 0.0001; Interaction *F*_(3,659)_ = 1.270, *p* = 0.2838), such that the increase mode in the high force condition was sustained longer throughout the trial and decreased closer to the decrease mode peak than the increase mode in the low force conditions, further supporting force dependence during static grasping in SC for this participant.

For dynamic conditions, we quantified when the increase mode reached 25–90% of its total modulation relative to the onset peak (Fig. 4*C*,*G*, top right). In P3, there was no significant effect of ramp duration, but there was a significant interaction between ramp duration and modulation level (Ramp Cond *F*_(3,1088)_ = 1.887, *p* = 0.1301; Mod Level *F*_(3,1088)_ = 163.2, *p* < 0.0001; Interaction *F*_(9,1088)_ = 2.400, *p* = 0.0108). Post hoc analyses indicated that ramp-related differences emerged only at the 90% level. Shorter ramps (1.5 and 3.0 s) which required faster force application reached late-phase recruitment earlier than longer ramps, whereas 4.5 versus 6.0 s ramps did not differ, indicating a plateau at longer durations. Thus, in P3, ramp duration influenced the onset → increase relationship only in the late phase of increase mode recruitment, consistent with a rapid early saturation followed by limited sensitivity to ramp duration. In P2, ramp effects emerged at higher modulation levels (75 and 90%)—late portions of the increase trajectory were delayed for longer ramps—while the 25 and 50% levels showed no reliable separation (Ramp Cond *F*_(3,944)_ = 12.79, *p* < 0.0001; Mod Level *F*_(3,944)_ = 177.4, *p* < 0.0001; Interaction *F*_(9,944)_ = 2.645, *p* = 0.005). Post hoc tests again showed that differences were driven by 1.5–3.0 s ramp conditions, with no significant difference between 4.5 and 6.0 s. Together, these results suggest that late-phase recruitment of the SC increase mode is rate dependent at shorter ramps but saturates at longer durations, with P2 showing more graded late-phase sensitivity (75/90%) and P3 showing earlier saturation, with effects only at the 90% level.

When aligned to the decrease peak, the increase mode consistently preceded the decrease mode (Fig. 4*C*,*G*, bottom right). In this comparison, we are specifically tracking the decay of the increase mode during the hold period leading up to release. In P3, this decay scaled robustly with ramp duration (Ramp Cond *F*_(3,1024)_ = 331.3, *p* < 0.0001; Mod Level *F*_(3,1024)_ = 50.91, *p* < 0.0001; Interaction *F*_(9,1024)_ = 2.836, *p* = 0.005), with shorter ramps showing faster decline and a plateau between 4.5 and 6.0 s. In P2, despite weaker overall modulation of the decrease mode, the increase → decrease relationship also showed significant ramp effects (Ramp Cond *F*_(3,934)_ = 280.4, *p* < 0.0001; Mod Level *F*_(3,934)_ = 35.59, *p* < 0.0001; Interaction *F*_(9,934)_ = 0.4029, *p* = 0.934), again driven by shorter ramps. Thus, across both participants, the decay of increase activity into release was stretched for longer ramps but plateaued at longer durations, mirroring the single-channel observation (Fig. 2*D*) that SC increase-tuned channels have longer response tails extending into the hold period.

Factor projections of the static grasping conditions along the most prominent SC modes showed a modest separation along the force-increase dimension, suggesting some encoding of attempted absolute force (Fig. 4*D*,*H*, left). This separation was weaker than the clear hold-related separation seen in MC, consistent with the absence of a dedicated hold mode in SC. Instead, the slow decay of the increase mode during static trials may provide a partial force representation. In the dynamic condition (Fig. 4*D*,*H*, right), P3 trajectories for fast and slow ramps were largely overlapping along the increase and decrease dimensions, providing little evidence for explicit encoding of ramp speed. For P2, interpretation was limited by the minimal modulation of the decrease mode, which reduced the dimensional separation of trajectories. This again contrasts with MC, where ramp timing effects were clearer, and supports the idea that SC contributes primarily to representing attempted absolute force levels rather than force rate dynamics.

Together with the latency analyses, these projection results reinforce that SC dynamics differ from MC: attempted absolute force is weakly represented through the increase dimension, while ramp trajectories lack strong separation by rate of force application. The increase mode's decay into the hold and release periods likely accounts for much of the static separation, underscoring its potential role in force encoding.

### Motor cortex encodes attempted absolute force during both static and dynamic grasping

**Figure 5.**
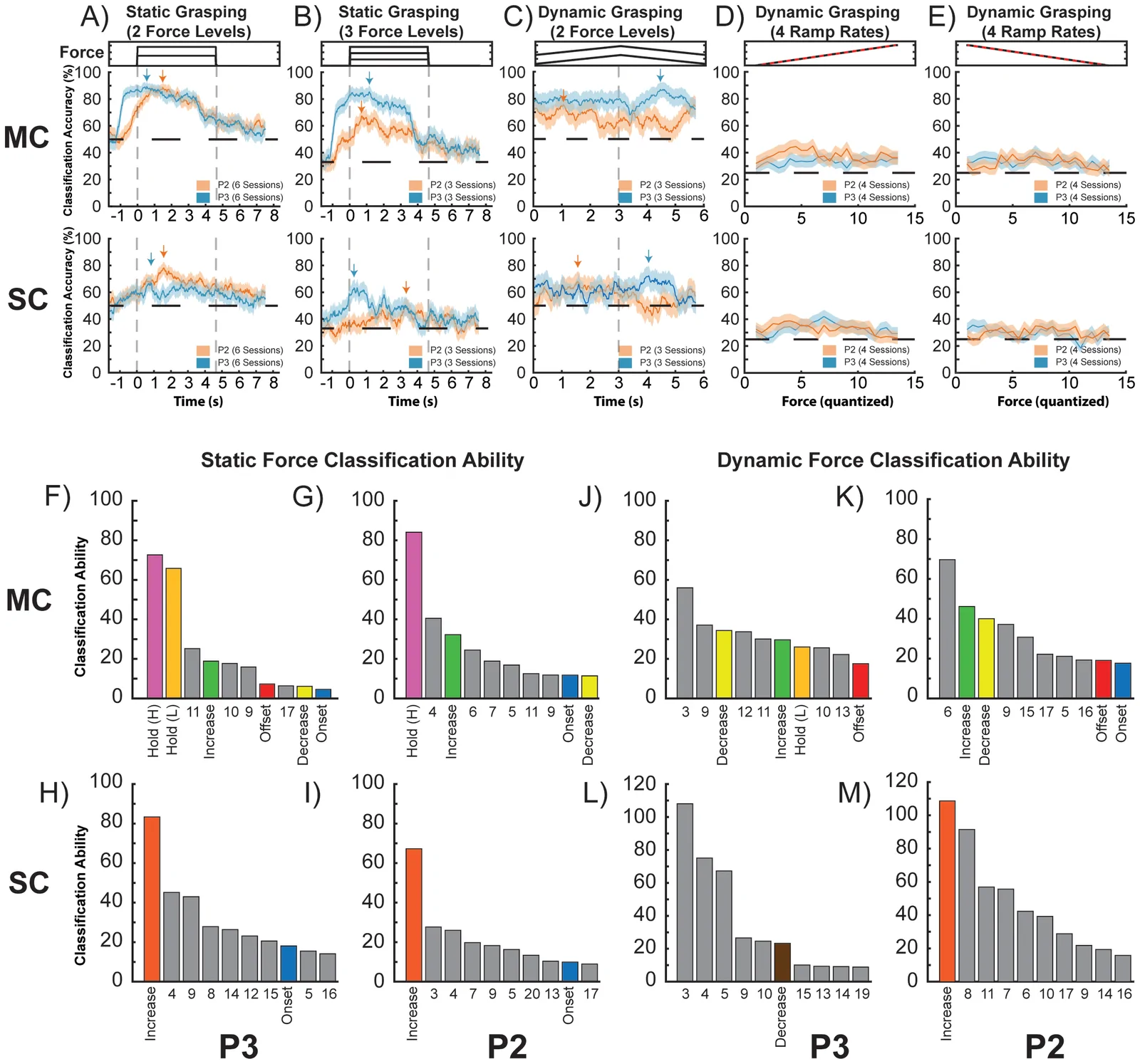
Force classification: ***A–C***, Force classification accuracies at each time point during static grasping with two force levels, static grasping with three force levels, and dynamic grasping (two force levels) in both MC and SC using a naive Bayes classifier. Arrows indicate the peak classification accuracy. Solid lines depict the mean classification accuracies and the shaded regions represent the variance over 10,000 bootstraps. ***D***, ***E***, Force ramping rate classification with four different ramps compared across force aligned windows using linear discriminant analysis (LDA) with uniform priors across four ramp-duration classes (1.5, 3.0, 4.5, and 6.0 s; chance = 0.25). ***F–M***, Classification abilities of individual neural modes in MC and SC for both participants. ***F–I***, Results of a two-force static grasping task similar to the classification results with all neural modes shown in ***A***. ***J–M***, Results of a dynamic grasping condition with two force levels similar to the classification results with all neural modes shown in ***C***.

We next asked how neural populations in MC and SC encode attempted force across static and dynamic conditions. Attempted force encoding was first evaluated using a naive Bayes classifier trained to discriminate force levels within each condition. In MC, classification accuracy for both two- and three-force levels was consistently above chance (Fig. 5*A*,*B*), peaking early in the hold phase and remaining elevated throughout much of the grasp before declining near offset. In SC, accuracies were lower than MC, peaking close to grasp onset for both participants in the two-force condition. For the three-force condition, peak accuracy occurred near grasp onset in P3 but later in the hold period in P2. These results indicate that both MC and SC carry force information during static grasps, with stronger and more sustained encoding in MC.

For dynamic grasping, we classified force conditions during the averaged dynamic periods of the sawtooth condition (i.e., ramp-up and ramp-down; Fig. 5*C*). Classification accuracies remained above chance level in MC for both participants throughout both the ramp-up and ramp-down phases. This peaked during the ramp-up phase for P2 and during the ramp-down phase for P3. However, the overall range of accuracies throughout the condition were lower than the static conditions. Like the static conditions, classification accuracies for the dynamic condition in SC were lower than in MC, and they remained relatively stable throughout the grasp for both participants.

We also asked whether neural activity distinguishes ramp rate (1.5, 3.0, 4.5, 6.0 s ramps to the same high attempted force target) after controlling for attempted absolute force. Rate decoding accuracy was slightly above chance for both participants and for both directions (Fig. 5*D*, ramp-up; Fig. 5*E*, ramp-down). Accuracy varied slowly with force, indicating that modest rate information is present across the force range rather than being confined to a specific level. Few individual bins reached significance—unsurprising given the modest per-bin effect sizes and multiple comparisons—but the aggregate pattern (median accuracy across bins and area-over-chance) was positive and similar across participants. These results show that while MC and SC contain some rate-related information, attempted absolute force encoding appears to be favored.

To understand how each individual neural mode contributes to attempted force encoding throughout the grasp, we retrained and tested a naive Bayes classifier using each neural mode as a single feature. We then calculated the classification ability of each mode by finding the area under the classification accuracy curve bound by the chance level on the *y*-axis and the relevant behavioral phase windows on the *x*-axis. This area was taken as a proportion of the area calculated when classifying using the full feature set. Effectively, classification ability is a measure of how well an individual neural mode can classify grasping force conditions. In MC during static grasping (two-force levels), the hold (high), hold (low), and increase-related neural modes had notable overall classification abilities for P3: 72.7, 65.8, and 18.9%, respectively. For P2, classification abilities for hold (high) and the increase-related modes were 84.1, and 32.2% (Fig. 5*F*,*G*). In SC, the increase mode had the highest overall classification ability for both participants: 83.4% for P3 and 67.2% for P2, which was most effective at classifying attempted force during the hold phase of the grasp (Fig. 5*H*,*I*).

During dynamic grasping (two-force levels), unlabeled MC modes #3 and #9 (Fig. S2*B*) had the greatest classification ability for P3 (Fig. 5*J*). The modulation patterns of these modes were similar to that of the decrease and offset modes during the low-force dynamic condition but were relatively inactive during high-force dynamic as well as static conditions, explaining their high classification abilities. Similarly, for P2, neural mode #6 (Fig. S2*D*), which had a modulation pattern resembling that of the offset mode only during the low-force dynamic condition, had the highest classification ability (Fig. 5*K*). This was followed by the force-increase and force-decrease modes. In SC during dynamic conditions, overall classification accuracies were low for both participants (Fig. 5*C*). For P3, neural modes #3–5 had the highest classification abilities but had inconsistent activation patterns across grasping conditions (Fig. 5*L*). For P2, the force increase mode had the largest classification ability (Fig. 5*M*).

Overall, during static grasp for both participants, the holding-related and increase-related modes were most informative of attempted force in MC, and the increase-related mode was most informative of attempted force in SC. During dynamic grasp, results were more varied. The increase- and decrease-related neural modes were informative of attempted force in MC for both participants, as was the increase-related mode in SC for P2.

### Shared MC–SC dynamics originate in motor cortex

**Figure 6.**
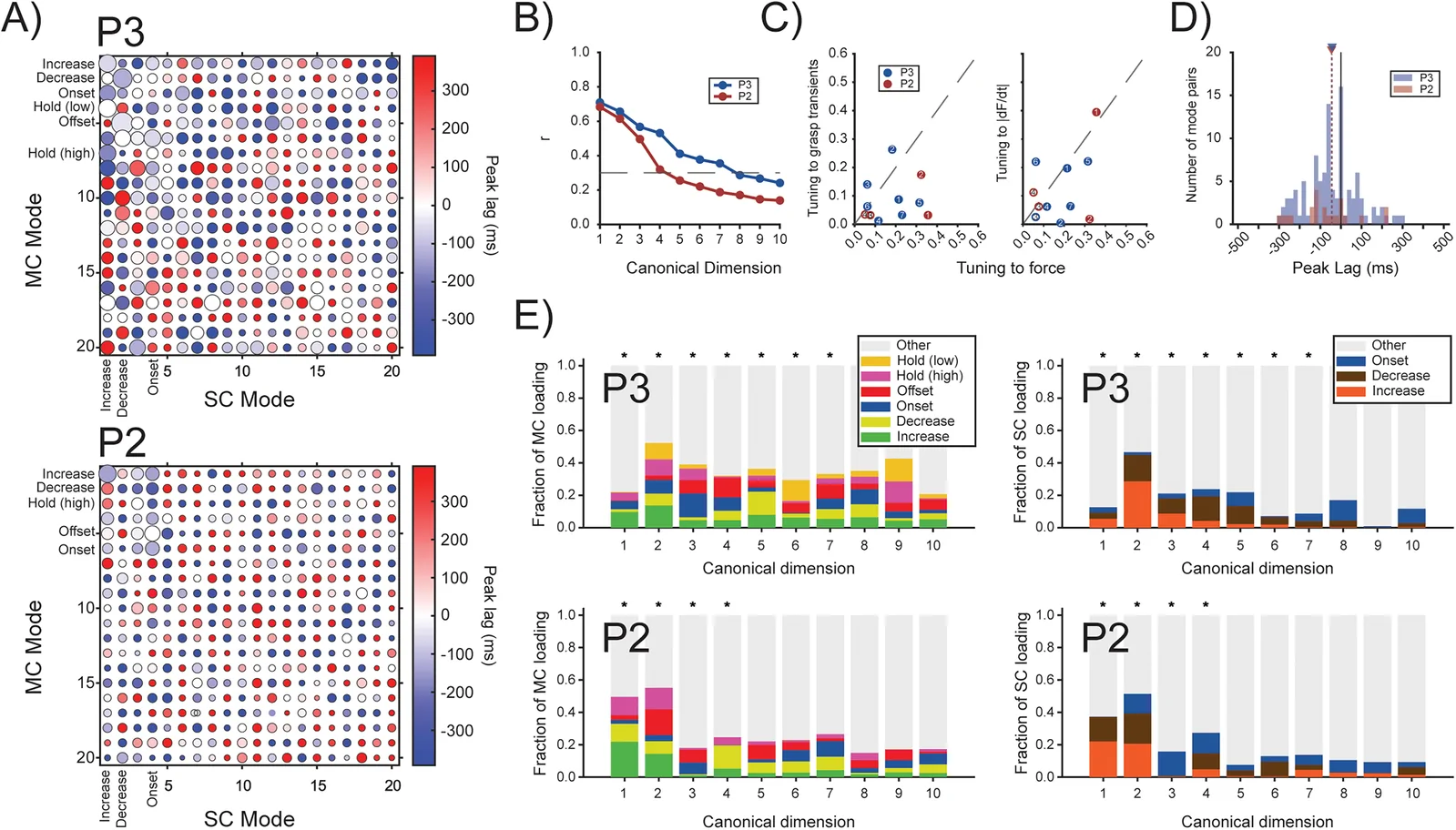
Canonical correlation analysis (CCA) of MC and SC population dynamics during attempted grasp. ***A***, MC × SC residual cross-covariance for every mode pair, computed after subtracting the per-condition trial-averaged time course from each factor (see Materials and Methods). Top, P3; bottom, P2. Marker size encodes |peak *r*| and color encodes the signed peak lag, with negative values (blue) indicating MC leads SC. Axes are ordered with labeled task-relevant modes first, followed by unlabeled modes by index. ***B***, Per-dimension canonical correlations (*r*) for each participant. The dashed line marks the *r* > 0.3 effect-size threshold used to define high-effect dimensions (7 in P3, 4 in P2). ***C***, Tuning of each high-effect canonical dimension to a continuous force command (*x*-axis, both panels) versus a cue-locked transient boxcar (left panel) or smoothed |dF / dt| (right panel). Markers are filled when at least one axis exceeds the participant-specific permutation null (see Materials and Methods); below the dashed *y* = *x* line, force-command tuning dominates. ***D***, Distributions of peak lags for mode pairs with nontrivial residual coupling (|peak *r*| ≥ 0.10). Triangles and dashed lines mark each participant's mean peak lag. Two-sided binomial sign tests: P3, *p* < 0.001 (*n* = 147, mean −43 ms); P2, *p* = 0.04 (*n* = 23, mean −46 ms). ***E***, Channel-loading composition for the top 10 canonical dimensions, broken down by labeled response class and an unlabeled “other” category. Top row, P3 MC (left), P3 SC (right). Bottom row, P2 MC (left), P2 SC (right). Asterisks mark high-effect dimensions. SC has no factors mapped to hold classes by construction.

To probe the latent communication structure between MC and SC, and to address whether the relative timing of MC and SC activity provides clues about the direction of signal flow, we performed CCA on the per-trial latent factor projections of MC and SC. We first asked whether the timing of shared dynamics carries information about the direction of signal flow. Trial-averaged structure shared by MC and SC can produce a spurious near-zero-lag correlation that masks any true directional asymmetry. To isolate trial-by-trial coupling, we subtracted the per-condition trial-averaged time course from each MC and SC factor before recomputing the canonical variates—analogous to noise correlation in signal/noise correlation analyses—and then computed the cross-covariance (linearly detrended; ±500 ms search window) for every MC × SC mode pair on these residual variates. The peak lag was recorded for each mode pair, and the sign of the peak lag was treated as a single observation per pair (negative = MC leads SC). The bubble panels in Figure 6*A* visualize this asymmetry across the full mode-pair matrix (marker size = |peak *r*|, color = signed lag). Restricting to mode pairs with nontrivial residual coupling (|peak *r*| ≥ 0.10), MC reliably led SC in both participants: in P3, the binomial sign test on *n* = 147 pairs gave *p* < 0.001 with a mean peak lag of −43 ms (Fig. 6*A*, top; Fig. 6*D*) and in P2, *n* = 23 pairs gave *p* = 0.04 with a mean peak lag of −46 ms (Fig. 6*A*, bottom; Fig. 6*D*).

We next asked whether the shared dynamics resemble a continuous motor command (corresponding to the attempted grasp force) or timing/transition-related signals. For each participant, we identified the canonical dimensions whose paired correlation between MC and SC variates exceeded *r* > 0.3 (a moderate effect-size threshold), yielding seven dimensions in P3 and four in P2 (Fig. 6*B*). Across these high-effect dimensions, the maximum canonical correlation reached *r* = 0.71 in P3 and *r* = 0.68 in P2 (Fig. 6*B*).

For each high-effect dimension, we computed the Spearman correlation between the paired canonical variates (averaging |*ρ*| across the MC and SC sides) and three trial-aligned regressors: the attempted force command, a grasp-transient boxcar (±500 ms around both the grasp-onset and release cues, capturing event-locked phasic structure around both transitions), and a force-transition regressor (smoothed |dF / dt|). Tunings were calibrated against a within-trial circular-shift permutation null (95th-percentile floor pooled across high-effect dimensions; per participant: P3 = 0.10, 0.07, 0.19 for force, grasp transients, and |dF / dt|, respectively; P2 = 0.15, 0.10, 0.37; the elevated |dF / dt| floor reflects the regressor's narrow temporal support relative to the variates' transient structure). Across high-effect dimensions, sustained-command tuning was modestly higher than event- or transition-locked tuning in most dimensions of both participants: in the command-versus-transients scatter (Fig. 6*C*, left), the majority of high-effect dimensions sat below the diagonal (5/7 in P3; 2/2 in P2) and a similar pattern held in the command-versus-transition scatter (Fig. 6*C*, right; 5/6 in P3, 1/2 in P2). Several individual dimensions exceeded their participant-specific noise floor for force-command tuning (most prominently P3 dim 5, mean |*ρ*| = 0.39; P2 dim 1, mean |*ρ*| = 0.40; and P2 dim 2, mean |*ρ*| = 0.35), but the absolute magnitudes of all tunings were modest, with no dimension exhibiting strong or selective alignment to any single regressor.

The factor-loading composition of these dimensions (Fig. 6*E*) was similarly mixed: most of the loading distribution fell on factors that we had not assigned to any labeled class (i.e., the “other” classification), with the labeled-class portion distributed across the increase, decrease, onset, offset, and hold classes. Hold-class contributions were small in MC and were not represented among labeled SC factors. We therefore interpret the loading composition cautiously and do not draw strong conclusions about the categorical identity of the shared subspace from this analysis alone.

Together, these results indicate that on a trial-by-trial basis, the shared fluctuations within the MC–SC subspace appear earlier in MC than in SC and that the subspace itself carries modest force-command-related structure alongside event- and transition-related signals.

## Discussion

### Summary of main findings

This study investigated cortical dynamics in motor (MC) and somatosensory cortex (SC) during static and dynamic grasp force tasks in participants with tetraplegia. Even without overt movement, attempted grasping engaged MC neurons and modulated SC neurons. At the single-channel level, MC exhibited transient and tonic responses tuned to grasp phases, while SC showed similar but less sharply tuned activity. At the population level, distinct neural modes emerged within each cortical area, modulated by grasp phase and informative of force condition. In MC, holding-related modes contribute most to force encoding, while SC modes were tuned to task phase and, to a lesser degree, force condition. Latency and force rate analyses indicated that both areas preferentially encoded attempted absolute force rather than rate of force.

### Response characteristics on the single channel level

Single-channel analyses revealed distinct transient and tonic firing patterns related to attempted grasping in MC, consistent with findings from intact primate motor systems performing precision grasps. Similar subpopulations have been observed across the motor cortex (Smith et al., 1975; Hepp-Reymond et al., 1978; Wannier et al., 1991; Maier et al., 1993; Hendrix et al., 2009), cerebellum (Smith and Bourbonnais, 1981), motor thalamus, globus pallidus (Anner-Baratti et al., 1986), and in spinal premotor interneurons (Takei and Seki, 2013), supporting consistency of force encoding motifs across the neuroaxis. We extend these results to attempted grasping in individuals with paralysis. Unlike earlier work, we did not observe extensive inhibition, which may reflect grasp type. Inhibitory responses appear more prominent during dexterous grasps requiring fine control (Wannier et al., 1991), whereas our participants performed attempted power grasps, which may engage broader excitatory populations. Indeed, grasp type interacts with force encoding across overlapping populations during both overt (Intveld et al., 2018) and attempted grasping (Rastogi et al., 2021). Phasic–tonic responses were also less common in our dataset, likely reflecting the absence of afferent feedback, which normally modulates MC through ongoing communication with SC (Tokimura et al., 2000; Lei and Perez, 2017; Osborn et al., 2021; Shelchkova et al., 2023; Bao et al., 2024).

### Somatosensory cortex activity without afferent input

SC, while classically viewed as sensory-driven, is also active during motor planning (Gale et al., 2021; Ariani et al., 2022), observation (Kuehn et al., 2014), and imagery (Jafari et al., 2020; Wandelt et al., 2022). fMRI studies have similarly shown that attempted finger movements in individuals with tetraplegia activate preserved SC representations (Kikkert et al., 2021; Downey et al., 2024). Our spiking-level data extend this evidence, revealing task-related modulation of SC neurons during attempted grasp. Given our experimental design, SC activity may reflect efference copy—internal motor signals that help distinguish reafference from external sensory events (Straka et al., 2018). Unlike the tonic SC responses typical of intact animals (Wannier et al., 1991), we observed elongated transient activity that extended into the holding phase, possibly reflecting sustained efferent signaling from MC. In intact systems, such signals might be masked by strong peripheral feedback, highlighting the advantage of the current paradigm for isolating cortical communication. In support of this, shared MC–SC activity appeared earlier in MC than in SC on the order of expected corticocortical conduction delays, in the direction predicted by the efference copy hypothesis, and consistent with recent ECoG findings (Gimple et al., 2026). However, a small consistent lag could in principle arise from indirect subcortical routes. The shared subspace contained modest force-command-related structure alongside event- and transition-related signals but was not dominated by any single signal type, consistent with SC activity partially reflecting an internal copy of MC’s output during attempted grasp.

### Distinct neural modes in both MC and SC

Population-level analyses revealed that MC activity decomposed into multiple distinct neural modes corresponding to functional task components. Transient modes were engaged during rapid force changes (onset and release), while tonic modes dominated during slower force modulation and sustained holds. Similar decompositions have been observed during both overt and imagined isometric wrist movements (Dekleva et al., 2024) and during isometric force tracing in nonhuman primates (Amematsro et al., 2025). Here we extend those findings to attempted static and dynamic grasping. While individual channels sometimes resembled these motifs, the population approach provides a complementary view, capturing mixed selectivity through shared loadings across modes and isolating orthogonal components of variance.

Dedicated holding-related modes indicate that MC contributes to sustaining attempted force, not just generating transients. Because our participants attempted rather than produced force, part of this sustained activity may reflect a maintained motor intention rather than the motor command and afferent feedback that ordinarily accompanies overt force maintenance. The present data cannot dissociate these possibilities, but a dedicated hold-related mode indicates that MC retains a representation of ongoing intended force. Noninvasive studies similarly report bursts of MC activity during dynamic force production but limited sustained activation during static hold (Thickbroom et al., 1999; Keisker et al., 2010; Flint et al., 2014; Jiang et al., 2020). Despite this, our hold-related modes carried the most information about attempted absolute force. Because MC populations largely encode overlapping kinetic and kinematic features, it remains unclear how “resilient” holding-related neural modes are to added motor complexities, such as during grasp-and-carry behavior. Previous iBCI work from our lab demonstrates that force decoding throughout hold is difficult (Downey et al., 2018), while decoding transient signals at grasp onset and offset can be robust (Dekleva and Collinger, 2025). Interestingly, we identified two hold-related modes in P3’s MC data, which reflect distinct excitatory subpopulations at the single-channel level. Channels driving the high-force-preferring mode showed clear positive monotonic force tuning, consistent with the classical characterization of identified corticospinal cells (Hepp-Reymond et al., 1978; Wannier et al., 1991), whereas channels driving the low-force-preferring mode formed a more distributed subpopulation with weaker, slightly low-force-biased modulation.

Unlike previous studies (Smith et al., 1975; Hepp-Reymond et al., 1978), we did not find robust MC encoding of force rate. We considered whether rate might be encoded by the speed of traversal along a shared low-dimensional path. However, the slow-ramp condition traced a partially self-overlapping path not present in the fast-ramp condition (Fig. 3*D*,*H*), and rate decoding from the full latent factor space remained only slightly above chance after controlling for absolute force (Fig. 5*D*,*E*), not consistent with a pure speed-based rate code.

Notably, we found distinct neural modes in SC during grasp and force modulation. Dimensionality in SC was lower than in MC, unsurprising given the lack of afferent input. Three neural modes were consistently modulated across sessions and conditions, independently associated with the onset of grasp, increases in force, and decreases in force. However, P2 displayed weak modulation of the decrease-related mode, consistent with the lack of classified force decrease transient channels in SC for that participant. Even in SC, we could classify force conditions above chance levels during both static and dynamic conditions in both participants. Classification accuracies were lower in SC than in MC, expected given the fewer recording and tuned channels in SC.

### Limitations

Several observations we describe as properties of MC or SC could reflect features of participant behavior or limits of our neural sampling. With no overt movement, we relied on cues to define phase timing. P3 appeared to initiate and release grasps earlier than cued, contributing to variable latencies. The fixed target presentations likely contributed to anticipatory activity. Differences in mode engagement between P2 and P3, such as saturation of ramp-related modulation at longer ramp durations, are compatible with inconsistent engagement over longer task phases. Further, SC was sampled with only 64 channels. Features we describe as absent in SC could therefore reflect undersampling, rather than true absence. Both participants had prior experience with grasping and force-modulation tasks (Downey et al., 2018; Dekleva et al., 2024; Dekleva and Collinger, 2025), though prior closed-loop work typically decoded grasp kinematics rather than force, and the ramp-and-hold paradigm was novel for both. We therefore consider it unlikely that the modes are primarily artifacts of feedback-driven training.

### Conclusion

Even in the absence of movement and somatosensory feedback, the human sensorimotor cortex retains rich representations of both transient and tonic phases of grasp force. MC and SC each engage distinct neural modes to handle changes in force versus holding steady forces, indicating partially independent control schemes. The approximate orthogonality of the modes suggests the utility of an iBCI that targets distinct subspace components for dynamic force decoding. A gated decoder that interpolates between these sources could transition smoothly between dynamic and static control phases, useful for tasks such as grasp-and-carry or graded object manipulation. We have implemented an effective similar gated decoding approach that leverages the onset and offset components to enable control of simple (low vs high force) static grasping (Dekleva and Collinger, 2025). The apparent separability of these force-related modes from kinematic representations raises the possibility that a dedicated force decoder could be trained and run alongside concurrent kinematic control, rather than competing for limited decoder bandwidth.
